# Pharmacokinetic, Clinical, and Myeloid Marker Responses to Acepromazine Sedation in Arabian Camels

**DOI:** 10.3389/fvets.2021.725841

**Published:** 2021-09-08

**Authors:** Mahmoud Kandeel, Adel I. Almubarak, Jamal Hussen, Wael El-Deeb, Katharigatta N. Venugopala

**Affiliations:** ^1^Department of Biomedical Sciences, College of Veterinary Medicine, King Faisal University, Al-Ahsa, Saudi Arabia; ^2^Department of Pharmacology, Faculty of Veterinary Medicine, Kafrelsheikh University, Kafrelsheikh, Egypt; ^3^Department of Clinical Scienses, College of Veterinary Medicine, King Faisal University, Al-Ahsa, Saudi Arabia; ^4^Department of Microbiology, College of Veterinary Medicine, King Faisal University, Al-Ahsa, Saudi Arabia; ^5^Department of Internal Medicine, Infectious Diseases and Fish Diseases, Faculty of Veterinary Medicine, Mansoura University, Manosura, Egypt; ^6^Department of Pharmaceutical Sciences, College of Clinical Pharmacy, King Faisal University, Al-Ahsa, Saudi Arabia; ^7^Department of Biotechnology and Food Science, Faculty of Applied Sciences, Durban University of Technology, Durban, South Africa

**Keywords:** acepromazine, camel, pharmacokinetics, flow cytometry, sedatives

## Abstract

Sedatives and tranquilizers are important in the control of excited camels during camel transport. This study was conducted to investigate the clinical sedation of camels with acepromazine and its correlation with pharmacokinetics and pharmacodynamics. The sedation score, heart rate, respiration, body temperature, and pharmacokinetics were monitored before and after acepromazine injection, and myeloid marker expression was analyzed using membrane immunofluorescence and flow cytometry. The distribution (t1/2α) and elimination (t1/2β) half-lives were 0.1 and 9.4 h, respectively. The volume of distribution at steady state (Vss) was 20.01 L/kg, and the mean residence time (MRT) was 12.25 h. Sedation started rapidly within 10 min followed by persistent low-medium sedation for 2 h with an average sedation score of 1.2 ± 0.61, which might be associated with a slow elimination phase and prolonged MRT. Compared to horses, camels showed a lower clearance rate, higher volume of distribution, and higher elimination half-life. Slight changes in body temperature and heart and respiratory rate, as well as a lower hematocrit and changes in blood cell composition, suggest the careful application of acepromazine in animals with abnormal blood parameters or poor vital conditions.

## Introduction

Camels are adapted to harsh and arid environments and constitute a major source of food, transport, and economic status in such areas. These animals are used in camel shows and races, which require long travel times, proper handling, and restraint. Under these conditions, sedation might be recommended to minimize stress, especially on the nervous and vigorous animals, and minimize the risk of injury or trauma during shipping. Sedation is a routine practice for active and aggressive camels undergoing clinical evaluations and surgical procedures in veterinary clinics (1).

Phenothiazine derivatives are commonly used in veterinary anesthesia as a sedative and tranquilizer due to their depressant action on the central nervous system (CNS) and the blockage of central dopaminergic receptors (2, 3). Acepromazine is the most commonly used phenothiazine derivative in veterinary medicine, used extensively to sedate horses and dogs. It depresses heart activity either through inhibition of the CNS or by direct action on the heart muscles, potentially leading to compensatory tachycardia (4). Moreover, acepromazine has also been associated with lower arterial blood pressure in horses (5), and its induction of hypotension and hemodynamic alterations can be reversed by norepinephrine infusion (6).

Although acepromazine decreases the required dosage of subsequently administered anesthetics, it may increase the risk of regurgitation in cattle (4). When used as a sedative and pre-anesthetic drug, it can also act as an antiemetic and antiarrhythmic (3) and, at a dose of 0.02 mg/kg, is preferred over xylazine in the sedation of pregnant cows in the late gestational stage due to lower stress on the fetus (7). Furthermore, acepromazine has been used at subclinical levels of <0.05 mg/kg to sedate horses during transport. The major premise beyond this application is that acepromazine sedates with limited loss of coordination or motor activity (8). However, doses above the recommended clinical level (0.15–0.30 mg/kg) have also been administered in equines (9).

The pharmacokinetics of acepromazine have been studied in several animal species, including dogs (10) and equines (11–14). In the urine of horses, acepromazine and its metabolites can be detected for up to 48 h post intravenous injection (12). Although the onset and the duration of acepromazine's effects in camels were 5 min and 2.3 ± 0.5 h, respectively (15), to our knowledge, the pharmacokinetics and pharmacodynamics of acepromazine administration in camels have not been assessed. There is an enormous need for long-lasting sedation of camels with minor effects on motor activity and coordination for traveling to races and handling during shipping.

The immunomodulatory effects of sedation have been described for multiple species (16). Most studies suggested a link between sedatives and the development of anti-inflammatory and immunosuppressive responses with increased susceptibility to infections (17, 18). For example, although acepromazine did not alter the blood parameters in cow calves (19) or cats (20), the hematocrit, white blood cell (WBC) count, red blood cell (RBC) count, and hemoglobin % were significantly decreased in horses at a dose of 0.05 mg/kg (9). Furthermore, in equines, acepromazine at 0.1 mg/kg showed anti-inflammatory and antioxidant properties by modulating equine neutrophil function (21). For the dromedary camel, the impact of acepromazine on the peripheral immune system has not been investigated.

The present study investigated the impact of acepromazine on leukocyte composition and the expression pattern of several immune cell markers in camels. The specific objectives of this study were to (1) address the pharmacokinetics and pharmacodynamics of intravenous administration of acepromazine in camels, (2) evaluate the clinical effect of intravenous 0.1 mg/kg acepromazine administration to camels during travel, and (3) monitor the response of the hematological parameters to acepromazine administration.

## Materials and Methods

### Animal Handling and Drug Administration

Six adult female Arabian camels were used in this study. The average body weight was 436.7 ± 84 kg, and the average age was 6.7 ± 0.8 years. The camels were reared at King Faisal University Camel Research Center (Al-Hofuf, Saudi Arabia). They were assessed for apparent health conditions and normal health indicators. Forty-eight hours before the experiment, feed was withdrawn and water was available *ad libitum*. The Ethics Committee of King Faisal University (approval no. KFU-REC/2020-03-02) approved all animal procedures. Acepromazine (Calmivet® 5 mg/ml) was obtained from Vetoquinol (Lavaltrie, Canada). Camels were injected intravenously into the right jugular vein with 0.1 mg/kg acepromazine. After acepromazine injection, a 14-gauge IV catheter (BD Biosciences; Franklin Lakes, NJ, USA) was aseptically introduced into the left jugular vein to collect blood samples. The catheter was flushed with a heparinized saline solution.

### Collection of Samples

Blood samples were collected for pharmacokinetic and flow cytometric analysis. For pharmacokinetic analysis, blood samples were collected in 5-ml vacutainer blood collection tubes with clot activator (BD Biosciences) immediately before acepromazine intravenous administration (T0) and 5, 10, 15, and 30 min and 1, 2, 4, 6, 12, and 24 h after injection. Blood samples were stored at room temperature for 20 min, with serum collected after blood centrifugation at 5,000 rpm for 15 min using a 2-16P centrifuge (Sigma Zentrifugen, Osterode am Harz, Germany). The serum was stored at −20°C in an MDF-U537D biomedical freezer (Sanyo, Osaka, Japan) until analysis. For flow cytometric analysis of blood leukocytes, whole EDTA blood was again collected immediately before acepromazine administration and 4 h after injection.

### Instrumentation for Liquid Chromatography

Chromatographic conditions were performed as previously described with slight modifications (13). High-performance liquid chromatography (HPLC) analysis of serum samples was performed using the Shimadzu LC-2030 system (Shimadzu, Nishinokyo, Japan) with a C18 monolithic column (50 × 4.6 mm). Elution was performed at a flow rate of 1.0 ml/min with isocratic solvents 0.25% acetic acid in acetonitrile and water (v/v, mobile phase A) (90:10). The injection volume was 10 μl. Detection was at 254 nm by ultraviolet spectroscopy. All data were analyzed using Lab Solution software (Shimadzu).

### Standard Solutions and Calibration

Acepromazine standard solutions (0.01. 0.1, 1, 10, 20, 40, and 80 ng/ml) (acepromazine maleate, >98% purity, Sigma-Aldrich, St. Louis, MO, USA) were prepared from standard stock (50 μg/ml) in ethanol. All solutions were filtered through a 0.45-μm membrane (Millipore, Burlington, MA, USA) before injection into the HPLC system.

### Extraction From Serum Samples and Analytical Technique Validation

Fifty microliters of each serum sample was added to 350 μl of acetonitrile (Sigma-Aldrich, St. Louis, MO, USA) to precipitate the proteins. Samples were mixed using a digital vortex mixer (Thermo Fisher Scientific, Waltham, MA, USA). The supernatant was collected after centrifugation at 13,000 rpm using a Multifuge X3R benchtop centrifuge (Thermo Fisher Scientific) for 10 min. The supernatant was evaporated under a nitrogen stream at 40°C, and the residue was dissolved in the mobile phase. The analytical technique was validated for selectivity, sensitivity, linearity, precision, accuracy, and stability. The analytical technique's selectivity was assessed by comparing the chromatograms produced from acepromazine standard samples to those obtained from blank samples. The lower limit of quantification (LLOQ) was used to determine the sensitivity of the analytical technique, with the LLOQ response being at least five times larger than the blank response at the acepromazine retention time. Different concentrations of acepromazine standard solutions were created to test the linearity of the analytical technique. The peak areas of these solutions were estimated, and the peak area vs. concentration of the standard solutions was used to create the calibration curve. The weighing regression of the calibration equation for acepromazine was estimated after plotting the obtained drug concentration vs. the peak area using the following equation: y = 1.7962x + 15.888. Intraday and interday precision were used to determine the analytical precision. Intra-assay precision and accuracy were calculated for six duplicates on the same analytical run at the LLOQ. Inter-assay precision and accuracy were measured after repeating the analysis in three independent analytical assays. The limit of detection (LOD) was assigned to the lowest concentration of the calibrator with a 3:1 signal-to-noise ratio (12). The limit of quantitation (LOQ) and LOD were assayed on different days and several times on the same day. The determined LOD and LOQ were 0.01 and 0.1 ng/ml, respectively. Recovery tests were used to determine the analytical accuracy. By comparing the amount of drug retrieved from the spiked serum samples with the actual added amount, the relative recovery of the drug was estimated. The percentage recovery was computed after three degrees of recovery were performed six times. The calibration curve showed good linearity between 0.1 and 40 ng/ml. The recovery rate was 89.6 ± 11.6, consistent with the previously recorded recovery rate (11, 12).

### Pharmacokinetic Analysis

The time–concentration relationship was processed by the non-linear curve fitting methods of the PKSolver Microsoft Excel add-in software (Redmond, WA, USA) (2). The data were empirically fit by non-compartmental and one-, two-, or three-compartment i/v bolus pharmacokinetic models. The two-compartment model optimally consolidated the obtained time–concentration results. The obtained pharmacokinetic parameters are provided in Table 1. The obtained pK-values are expressed as mean ± SD.

**Table 1:** The pharmacokinetic parameters for acepromazine (0.1 mg/kg) after intravenous administration in camels (*n* = 6).

**Parameter**	**Unit**	**Value (mean)**	**SD**
A	μg/ml	35.59	4.63
α	1/h	6.71	0.85
B	μg/ml	3.67	0.56
β	1/h	0.08	0.03
k_10_	1/h	0.77	0.25
k_12_	1/h	5.31	0.62
k_21_	1/h	0.71	0.15
t_1/2α_	h	0.10	0.01
t_1/2β_	h	9.40	2.94
C_0_	μg/ml	39.26	4.87
V	L/kg	0.2600	0.0300
CL	L/hr/kg	0.1900	0.0400
V_2_	L/kg	20.00	1.10
CL_2_	L/hr/kg	10.00	0.80
AUC_0−t_	μg/ml^*^h	44.56	5.05
MRT	h	12.25	4.1
V_ss_	L/kg	20.01	1.1

*α, the apparent fast rate constant; β, the apparent slow rate constant; A, the intercept of the α phase at y-axis; B, the intercept of β phase at y-axis; t_1/2α_, the distribution half-life; t_1/2β_, elimination half-life; V, the central compartment volume; V_2_, the second compartment volume; Vss, the volume of distribution at steady state; MRT, the mean residence time; Cl, total serum clearance; Cl_2_, clearance from the second compartment; AUC_0−t_, area under curve up to the last measurable concentration*.

### Clinical Evaluation

Camels were restrained manually in sternal recumbency before an initial physical examination was performed. Baseline sedation score, heart rate (using a stethoscope), respiratory rate (counting thoracic movements), and rectal temperature (electronic thermometer) were assessed. Electrocardiogram recording started immediately after acepromazine premedication, using a patient monitor (LifeVet M Eickemeyer, Tuttlingen, Germany). The temperature, respiration, and heart rate were monitored throughout the experiment. Parameter values were recorded again at 5 and 10 min after acepromazine premedication and then every 10 min after 90 min.

Sedation was scored on a 4-point scale (0 = no sedation with normal movement; 1 = mild sedation, slightly decreased movement, and reduced eye alertness; 2 = moderate sedation, moderately decreased movement, and resistance to handling; 3 = deep sedation, markedly decreased movement, and no resistance to handling) (22). Two blinded assessors who were unaware of the experimental design but familiar with camels' normal behavior assessed sedation and response to nociceptive stimuli through the study.

### Hematological Parameters

Samples for hematological evaluation were performed by aspiration of 5 ml of blood from the left jugular vein before and 4 h after acepromazine injection. Four hematological parameters were used to assess the pharmacodynamic effects of acepromazine in camels. The samples were analyzed by the scil Vet abc Plus veterinary hematology analyzer (Scilvet, Ontario, Canada). The machine was maintained and calibrated according to the manufacturer instructions.

### Immunofluorescence and Flow Cytometry Studies

#### Separation of Blood Leukocytes

Complete camel leukocytes were isolated after hypotonic lysis of blood erythrocytes (23). Stimulated blood samples suspended in phosphate-buffered saline (PBS) (Sigma-Aldrich, Steinheim, Germany) were centrifuged at 4°C for 10 min at 1,000 *g* using a Multifuge X3R benchtop centrifuge (Thermo Fisher Scientific), as the cell pellet was suspended in distilled water (Eppendorf, Hamburg, Germany) for 20 s, with double-concentrated PBS added to restore tonicity. These procedures were repeated for complete erythrolysis. Separated cells were suspended in MIF buffer (PBS containing bovine serum albumin (5 g/L) and NaN_3_ (0.1 g/L) (Sigma-Aldrich) at 5 × 10^6^ cells/ml.

#### Membrane Immunofluorescence and Flow Cytometry

Membrane immunofluorescence and flow cytometry were used to examine the expression of several myeloid markers (24). Separated leukocytes (4 × 10^5^) were incubated with unlabeled primary monoclonal antibodies (mAbs) specific for the cell surface molecules, CD4, WC-1, CD14, CD163, and MHCII (Kingfisher Biotech, St. Paul, MN, USA) (23). After treatment (15 min, 4°C), cells were washed twice and treated with mouse secondary antibodies IgM, IgG1, and IgG2a (Invitrogen, Carlsbad, CA, USA) tagged with different fluorochromes. Mouse isotype control antibodies (BD Biosciences) were also included. Washed cells were analyzed with the Accuri C6 flow cytometer (BD Biosciences). At least 100,000 total leukocytes were collected and analyzed with Cflow software, version 1.0.264.21 (BD Biosciences, San Diego, CA, USA).

### Statistical Analyses

Statistical analyses were completed using the software Prism (GraphPad software version 9, San Diego, CA, USA). Results are expressed as a median. Quantitative results with non-parametric distribution of Levene's test were conducted using the Mann–Whitney test to compare two groups, comprising comparison hematological and myeloid markers before and after acepromazine treatment. Kruskal–Wallis was used to compare groups of three or more with Duncan test variance analysis to reveal the significance of the measured clinical parameters at different time points in comparison with the initial value. The results were presented as a boxplot for comparison between groups at different periods (*N* = 1) and linear for the median of camels (*N* = 6) regarding time degree. A *p*-value of <0.05 was considered significant (25).

## Results

### Pharmacokinetic Analysis

**Figure 1:**
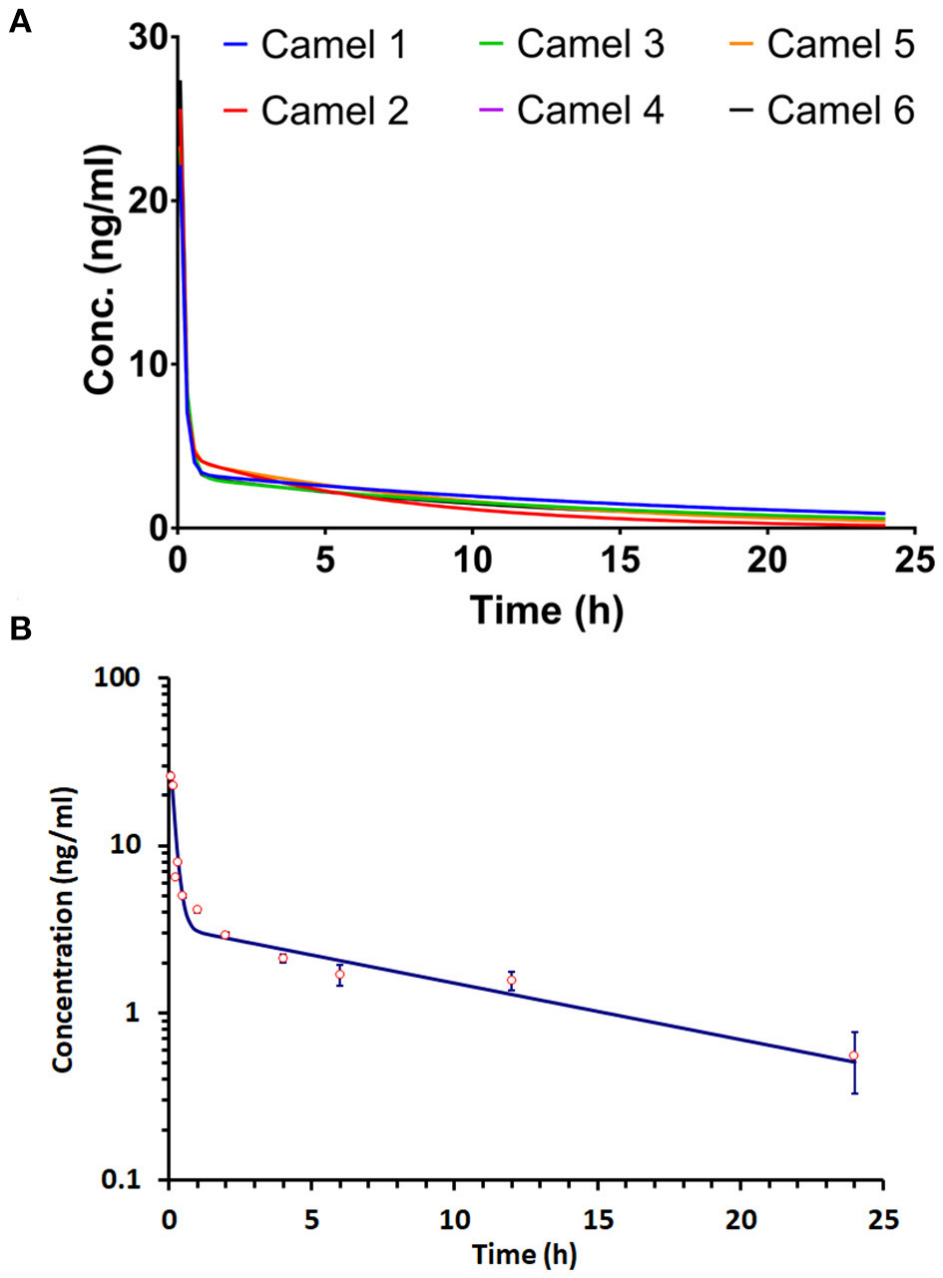
Progression of the acepromazine concentration–time curve after 0.1 mg/kg i.v. administration in Arabian camels (*n* = 6). **(A)** The time–concentration curve progression in individual camels. **(B)** The mean ± SD of time–concentration values for all camels.

The concentration–time progression curve for i.v. acepromazine administration (0.1 mg/kg) is provided in Figure 1. The curve shows a rapid decline corresponding to the distribution phase followed by a slower decline for the elimination phase. The one-compartment model was rejected as the curve did not display a monotonic decline. The best fit of data was obtained by non-linear fitting, and the two-compartment model best described acepromazine disposition in camels.

The apparent fast (α) and slow (β) rate constants were 6.71 and 0.08 h^−1^, respectively. The distribution (t_1/2α_) and elimination (t_1/2β_) half-lives were 0.1 and 9.4 h, respectively. The central compartment volume (V) was 0.26 L/kg. The peripheral compartment volume was 20 L/kg. The volume of distribution at steady state (Vss) was 20.01 L/kg. The mean residence time (MRT) was equal to 12.25 h.

### Clinical Evaluation

**Figure 2:**
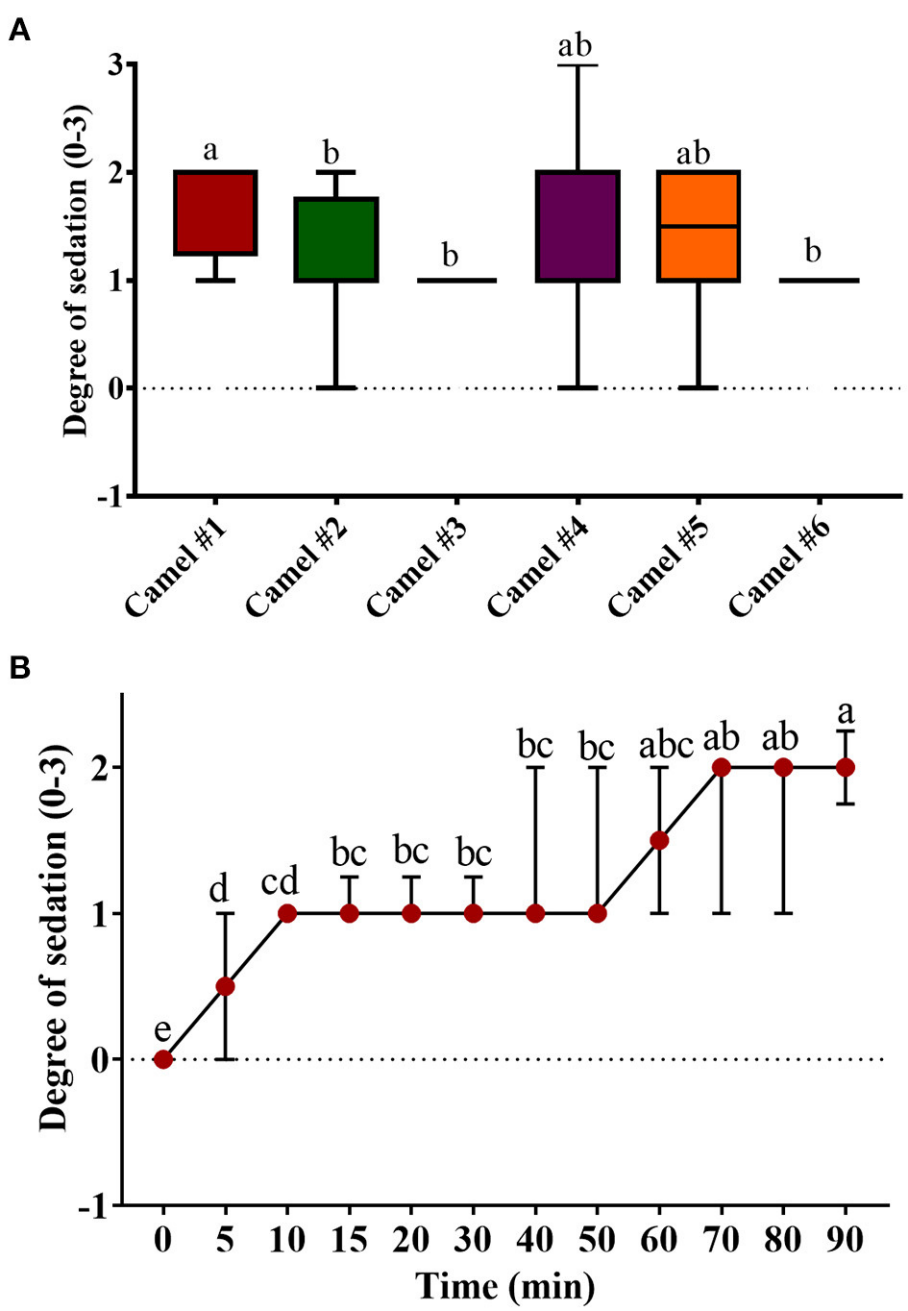
The sedation score in camels after injection of acepromazine (0.1 mg/kg i.v.). **(A)** Boxplot showing sedation scores with all data points represented. **(B)** The progression of acepromazine sedation in camels. Each data point represents the average of two recordings from two experts for all camels. Median values in each plot followed by a different lowercase letter (a, b, c) are significantly different by the Duncan test at *p* ≤ 0.05.

**Figure 3:**
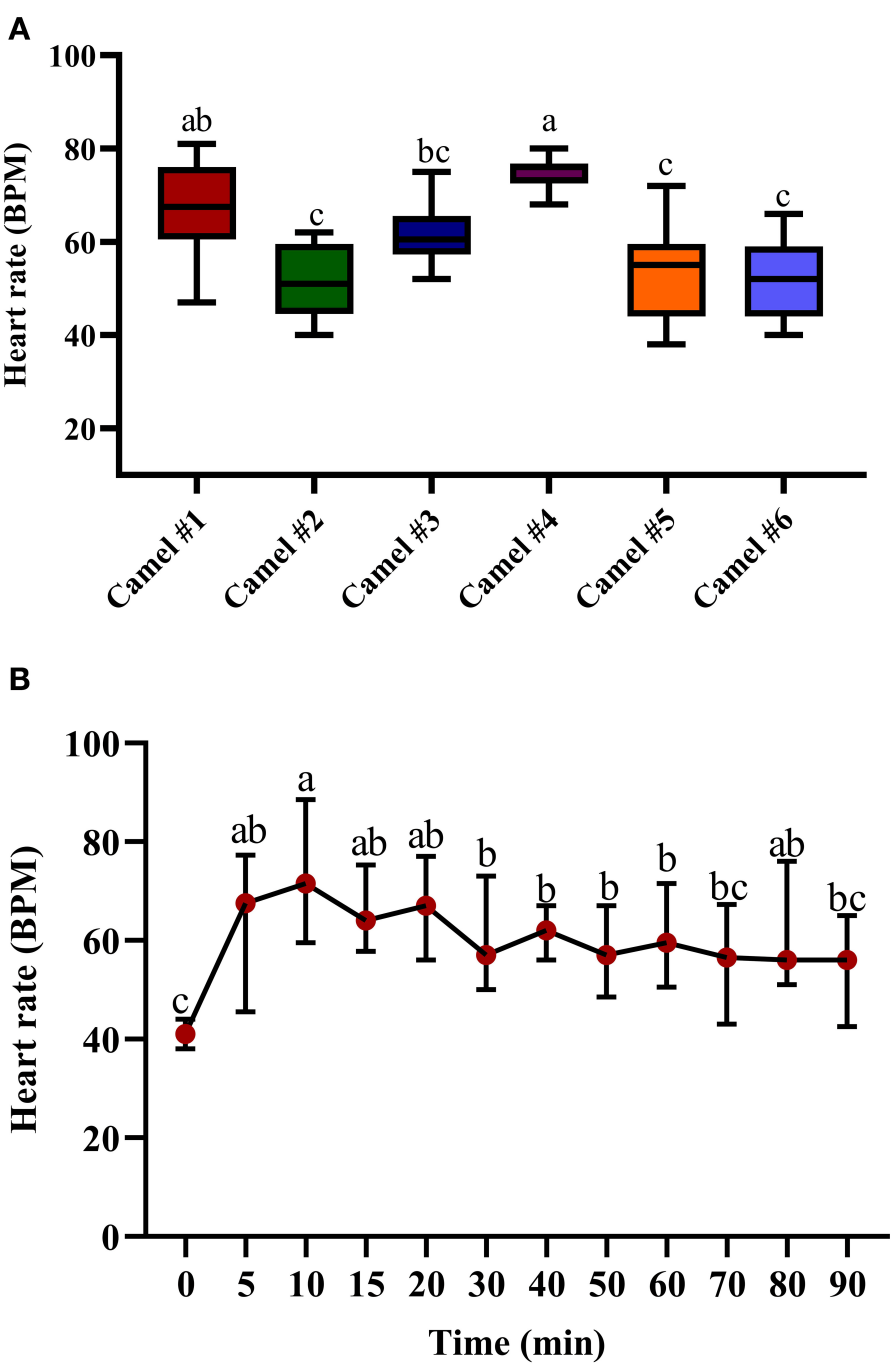
The changes in heart rate (beats/min) in camels after injection of acepromazine (0.1 mg/kg i.v.). **(A)** Boxplot showing heart rate (beats/min) values with all data points represented. **(B)** The median heart rates 90 min after acepromazine injection. Median values in each plot followed by a different lowercase letter (a, b, c) are significantly different by the Duncan test at *p* ≤ 0.05.

The clinical exam consisted of tracing the sedation, heart rate, respiratory rate, and changes in rectal temperature. Figures 2, 3 summarize the obtained clinical exam parameters. The sedation scores recorded for individual camels are provided in Table 2.

**Table 2:** The sedation scores after acepromazine (0.1 mg/kg) intravenous administration in dromedary camels (*n* = 6).

**Time (min)**	**Camel #1**	**Camel #2**	**Camel #3**	**Camel #4**	**Camel #5**	**Camel #6**
0	0	0	0	0	0	0
5	1	0	1	0	1	0
10	1	1	1	1	1	1
15	2	1	1	1	1	1
20	2	1	1	1	1	1
30	2	1	1	1	1	1
40	2	1	1	1	2	1
50	2	1	1	1	2	1
60	2	1	1	2	2	1
70	2	2	1	2	2	1
80	2	2	1	2	2	1
90	2	2	1	3	2	2

Figure 2 shows the tracing of sedation in camels and the progression of sedation during the experiment. The sedation score in camel #1 was significantly greater than in all other camels (Figure 2A). The lowest sedation scores were obtained for camels #3 and 6. Sedation started soon after acepromazine injection, with depression observed within 10 min after injection. Mild sedation was observed between 5 and 50 min after acepromazine administration and gradually increased toward moderate sedation. The maximal effect occurred at 70 min after treatment (Figure 2B). Muscle relaxation and the dropping of the lower lips were observed after 30 min in all the animals and persisted for 2 h. Lateral recumbency was observed in three camels with marked unconsciousness and occasional snoring, occurring after 30 min in two camels and after 90 min in the third camel, which persisted for several minutes. After lateral recumbency, the camels returned to a sitting position, with marked depression with the extension of the neck across the ground. Most camels had sedation scores of 1–2 (Figure 2A), and only one camel showed a sedation score of 3 after 70 min. In addition, there were significant differences in the sedation between individual camels (Figure 2A).

The tracing of the heart rate after acepromazine injection is provided in Figure 3A, with significant differences between individual camels. The initial recorded median heart rate was 41.65 (Figure 3B). The traced changes over time indicate a gradual increase in heart rate, reaching the maximal value after 10 min (69.65) with statistically significant tachycardia between 10 and 20 min after acepromazine injection (*p* < 0.05, Figure 3B). After 10 min, the heart rate gradually declined, reaching 56.42 after 90 min (Figure 3B). There was an initial increase in heart rate in all the camels soon after acepromazine injection, which did not return to its basal level within 2 h after injection.

**Figure 4:**
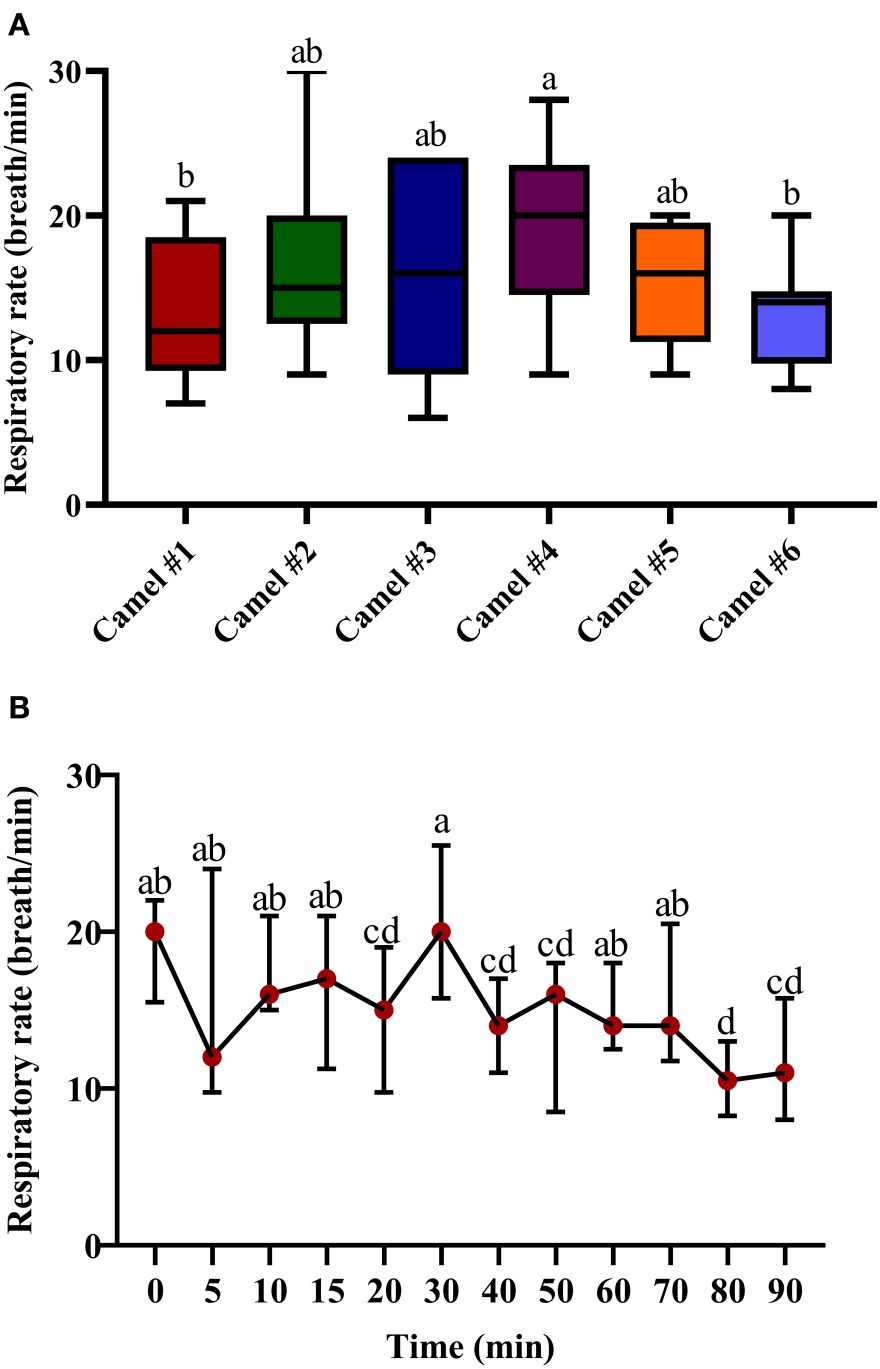
The changes in the respiratory rate (breath/min) in camels after injection of acepromazine (0.1 mg/kg i.v.). **(A)** Boxplot showing respiratory rates (breath/min) with all data points represented. **(B)** The median respiratory rates (beats/min) 90 min after acepromazine injection. Median values in each plot followed by a different lowercase letter (a, b, c) are significantly different by the Duncan test at *p* ≤ 0.05.

Figure 4A displays the data points of the minimal, maximal, and main concentration of the respiratory rate; the displayed cross lines within the boxes indicate the median values for each camel. The initial median respiratory rate was 19.47 (Figure 4B). The traced changes in respiratory rate over time indicate a gradual decrease in respiratory rate, although a temporary and brief rise in respiratory rate was observed at 30 min post injection of acepromazine. At 90 min, the respiratory rate was 13.54 (Figure 4B).

**Figure 5:**
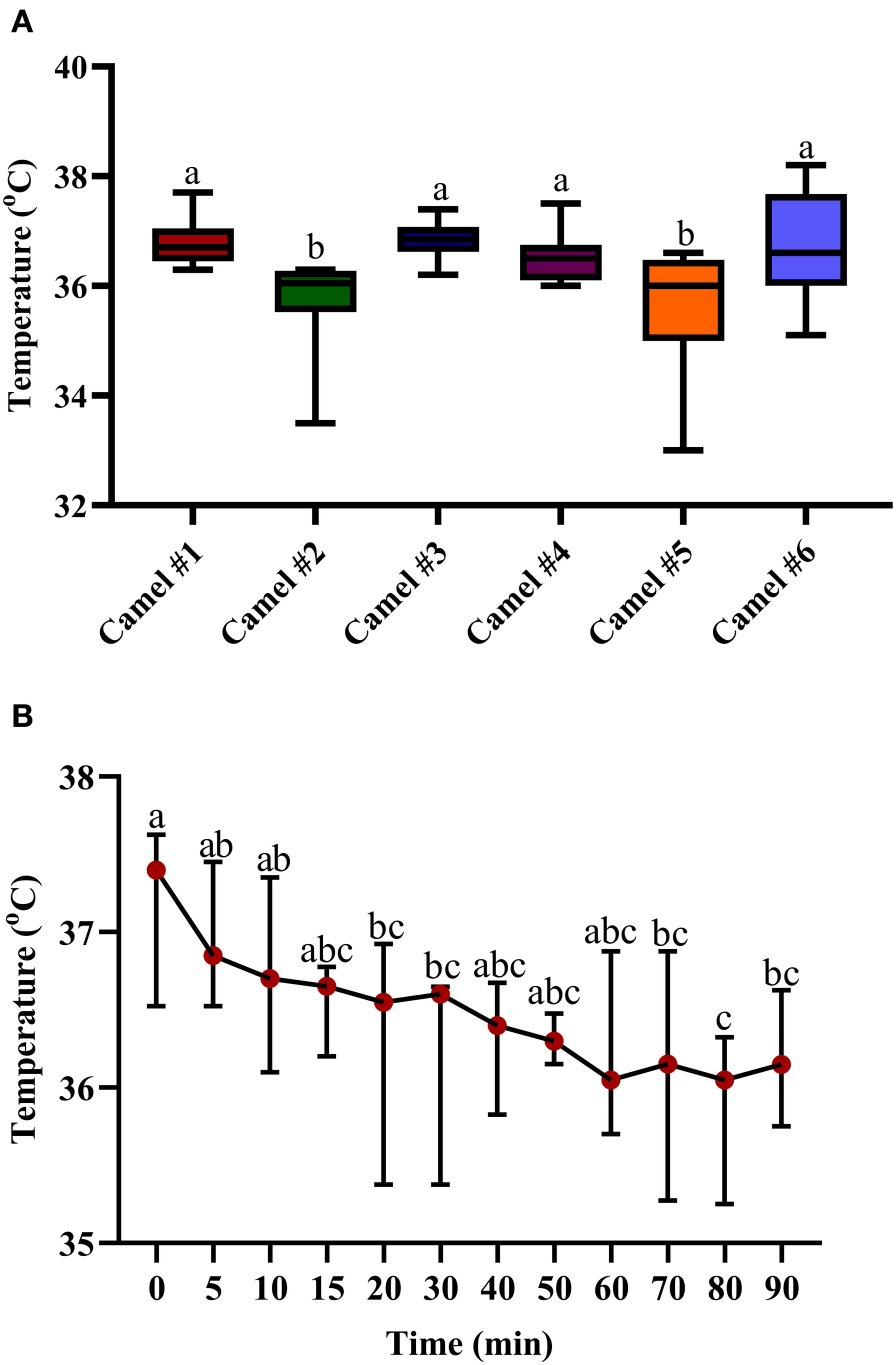
The changes in body temperature (°C) in camels after injection of acepromazine (0.1 mg/kg i.v.). **(A)** Boxplot showing body temperatures (°C) with all data points represented. **(B)** The median body temperatures (°C) 90 min after acepromazine injection. Median values in each plot followed by a different lowercase letter (a, b, c) are significantly different by the Duncan test at *p* ≤ 0.05.

Figure 5A displays the minimal, maximal, and main concentration of the recorded temperature; the displayed cross lines within the boxes indicate the median values for each camel. The initial median temperature was 37.61°C (Figure 5B). The traced changes in temperature over time indicate a gradual decrease in temperature. At 90 min, the temperature was 36.42°C (Figure 5B). The measured hypothermia was statistically significant at 15, 20, and 40 min after acepromazine injection (*p* < 0.05, Figure 5B).

### Hematological Parameters

Table 3 shows the obtained blood parameters before and 4 h after acepromazine injection. RBC count, hematocrit, and hemoglobin significantly decreased after 4 h (*p* < 0.05).

**Table 3:** The hematological parameters of camels after injection of acepromazine (0.1 mg/kg i.v.).

		**Before treatment**	**After acepromazine injection**	***p*** **-values**	**Normal range**
RBC	10^6^/mm^3^	8.71	6.87	0.031^*^	6.8–12.9
HGB	g/dl	12.57	9.95	0.015^*^	11–19
HCT	%	30.60	19.80	0.034^*^	30–58
PLT	10^3^/mm^3^	245.50	230.00	0.37^ns^	100–250

*The samples were collected twice: before treatment and 4 h after treatment.*

* *significant at p-values < 0.05; ns: non-significant at p-values >0.05*.

### The Impact of Acepromazine on Leukocyte Composition in Camel Blood

**Figure 6:**
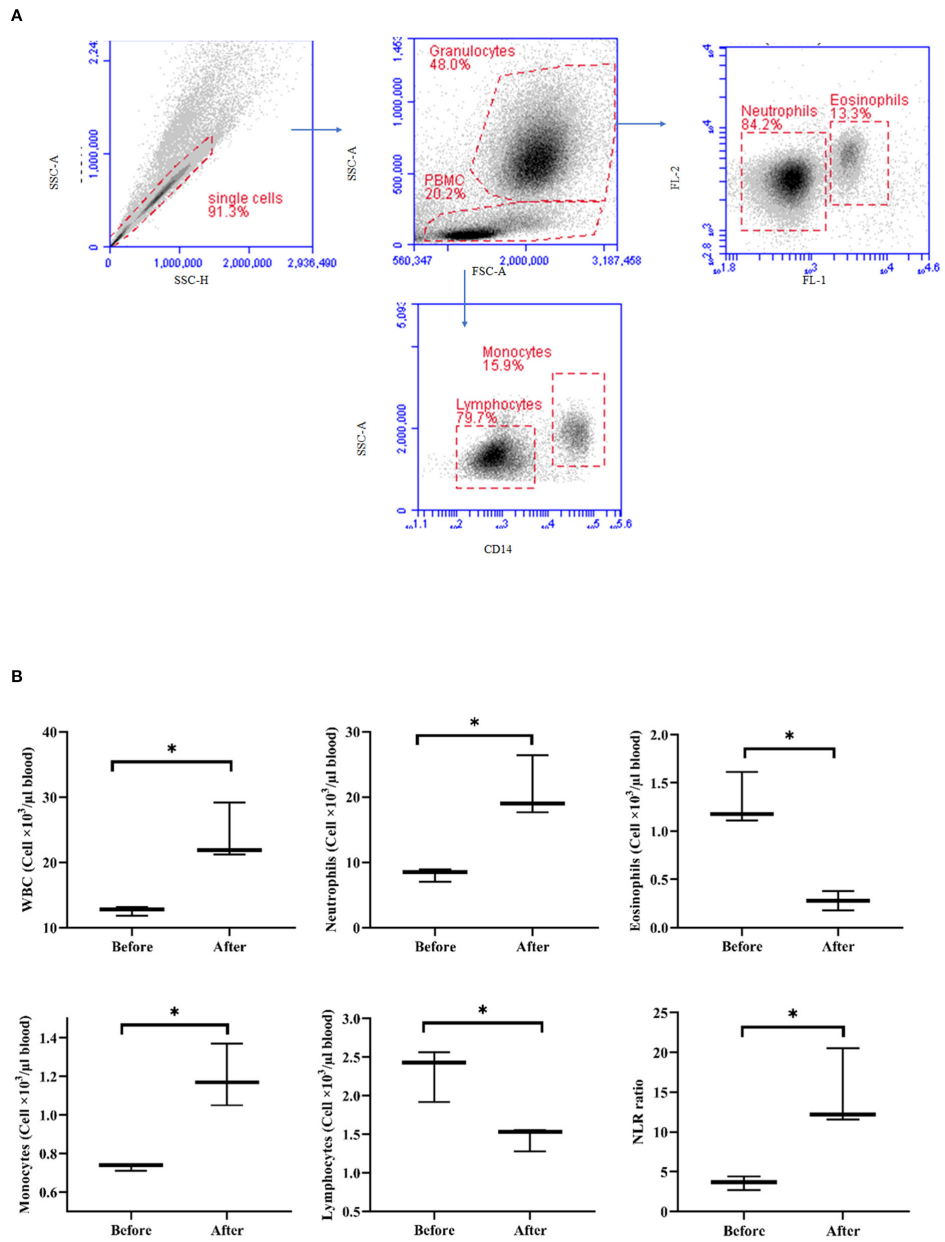
Impact of acepromazine on leukocyte composition in camel blood. **(A)** Single cells were excluded in the side scatter height (SSC-H) against SSC-Aria (SSC-A) plot. In the SSC-A against forward scatter (FSC)-A dot plot, camel granulocytes, and mononuclear cells were gated based on their scatter characteristics. Based on their different autofluorescence in FL-1, camel neutrophils, and eosinophils were identified. Camel monocytes and lymphocytes were identified as CD14-positive and CD14-negative mononuclear cells, respectively. **(B)** Boxplot showing whole WBC. Median cell numbers are presented for all leukocyte subsets before and after treatment. Differences between the median were calculated using the Mann–Whitney test and were considered significant (*) if *p* < 0.05.

After treatment with acepromazine, the total WBC counts significantly increased (*p* < 0.05) relative to the WBC counts before treatment (Figure 6). The differential counting of camel leukocyte populations revealed significantly (*p* < 0.05) more neutrophils and monocytes after treatment with acepromazine than before treatment. The number of eosinophils and lymphocytes, however, decreased significantly after treatment. The treatment-induced increase in neutrophil numbers, together with the decrease in lymphocyte numbers, resulted in a significantly higher neutrophil-to-lymphocyte ratio (NLR) in camel blood after treatment with acepromazine than that before treatment (Figure 6).

significant different between the median values

### Lymphocyte Composition in the Blood of Acepromazine-Treated Camels

**Figure 7:**
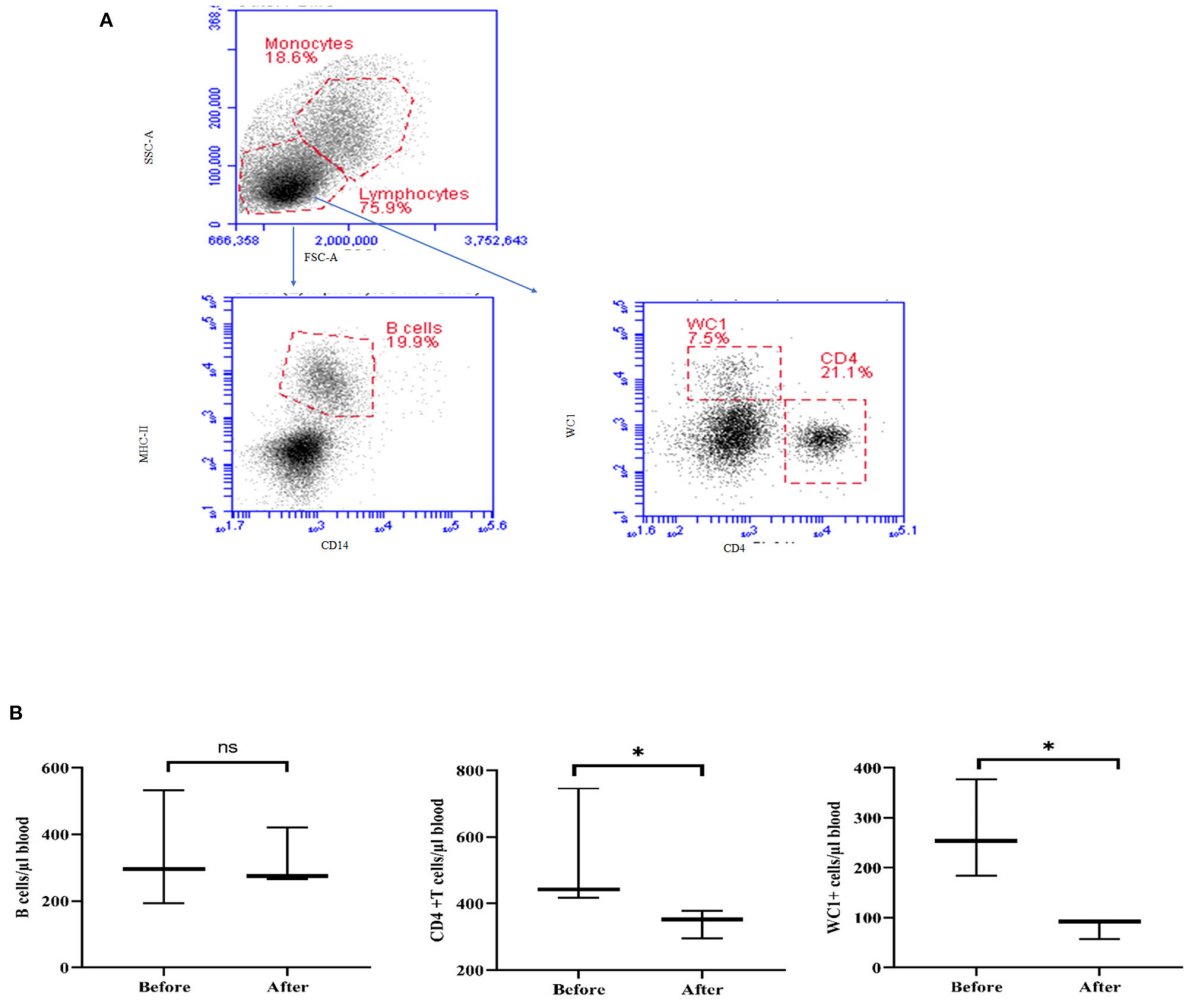
Impact of acepromazine on lymphocyte composition in camel blood. **(A)** After gating on lymphocytes within the mononuclear cell population in the SSC-A/FSC-A dot plot, camel B cells were identified as MHC-II+CD14– cells in the CD14/MHC-II dot plot. CD4+ T cells and gd T cells were identified based on their single staining with CD4 and WC-1, respectively. **(B)** The numbers of camel B cells, CD4+ T cells, and gd T cells were estimated by multiplying their percentages in blood lymphocytes by the absolute number of lymphocytes. Median cell numbers are presented for all leukocyte subsets before and after treatment. Differences between the medians were calculated using the Mann–Whitney test and were considered significant (*) if *p* < 0.05, ns denotes non-significant at a *p*-value >0.05.

Treatment with acepromazine induced a significant change in lymphocyte composition (Figure 7). Compared to their cell numbers before treatment, the number of CD4+ T cells and WC-1+ T cells decreased significantly in the blood of camels after treatment with acepromazine, while the number of B cells did not change (Figure 7).

### Acepromazine Modulates the Phenotype of Blood Monocytes

**Figure 8:**
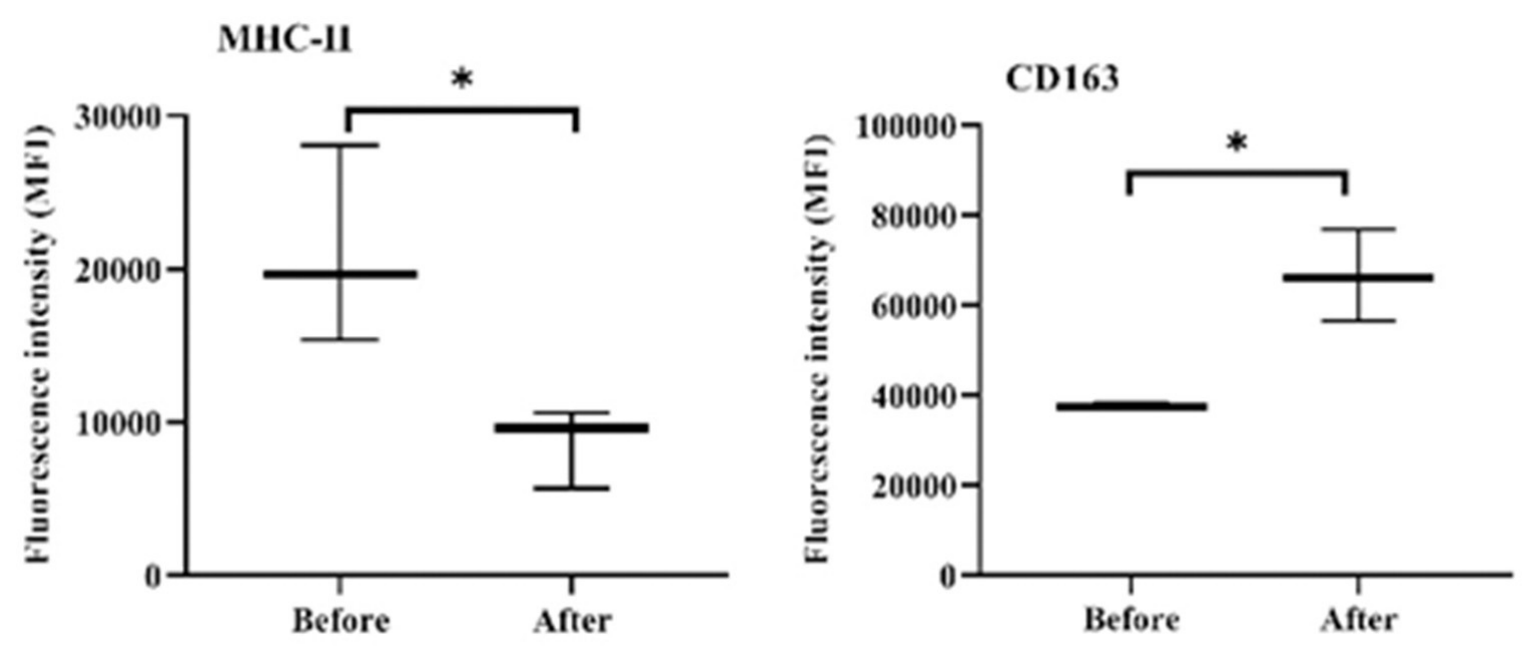
Expression densities of the cell surface molecules MHC-II and CD163 on camel monocytes. Monoclonal antibodies to CD14, MHC-II, and CD163 were used to mark separated camel leukocytes, and the labeled cells were examined by flow cytometry. The expression levels of MHC-II and CD163 were evaluated as MFIs of the investigated markers after gating on camel monocytes (based on their positive staining with CD14). *indicates a significant difference (*p*-value < 0.05) between groups as analyzed by the Mann–Whitney test.

The expression density (median fluorescence intensity, MFI) of the cell markers MHC-II and CD163 on blood monocytes was significantly changed after acepromazine treatment (Figure 8). Furthermore, monocytes significantly decreased their surface MHC-II expression, while they increased their CD163 expression relative to their basic expression values before treatment (Figure 8).

## Discussion

In this study, we present the first investigation of the hematological and cellular responses, clinical effects, and pharmacokinetics of acepromazine sedation in camels. By critically evaluating the sedation scheme and progression of sedation, camel practitioners can assess the potential application of acepromazine during surgical operations, long travel, and shipping.

We selected an acepromazine dose rate of 0.1 mg/kg, a dosage used previously in camels (15). A dose range from 0.02 to 1.5 mg/kg has been widely used for sedating animals. For example, the acepromazine dosage for animal tranquilization was 0.15 mg/kg in yaks (26), 0.02 mg/kg in pregnant cattle (7), 0.05 mg/kg in cattle (27), and 0.08–0.1 mg/kg in sheep (28, 29). A t_1/2α_-value of 0.438 ± 0.02 h was obtained in horses (12), higher than that found for camels (0.1 h).

Although the best fit of pharmacokinetic data in camels was a two-compartment pharmacokinetic model, the same as in horses (13, 14), the camels showed smaller t_1/2α_, higher t_1/2β_, a larger volume of distribution, slower clearance, and a longer MRT. In horses, t_1/2β_ has been measured at 5.16 h in thoroughbreds administered 0.09 mg/kg intravenously (13). In camels, the estimated t_1/2β_ was 9.4 h, almost double that in horses. In addition, camels showed a substantially higher MRT than horses, which has been previously estimated to be up to 50 h in serum and 111.6 h in urine of horses (9), while the Vss was 5 L/kg (14). These values are much lower than the estimated MRT and Vss of acepromazine in camels (Table 1). The volume of distribution (V2) was higher in camels than in horses, indicating greater distribution to tissues. Moreover, camels showed lower clearance and higher AUCs and Vd-values. These data indicate an observed late response to acepromazine's tranquilizing effects.

The average sedation score was 1.2 ± 0.61, with a markedly prolonged sedation time of several hours, in contrast to horses and cow calves, which experience a rapid onset and rapid course of sedation. Cow calves experienced a rapid onset of sedation for a short duration, becoming ataxic after 14.5 min, completely recumbent within 21 min, and recovered after 86 min (19). The peak sedation effect in camels did not correlate with the acepromazine serum concentration in camels, as the maximal sedation was observed after 90 min, which coincided with only 13.8% of the maximal value of serum concentration. However, the larger volume of distribution, slower clearance, and longer MRT could be associated with the observed clinical prolonged sedation score within the first and second score ranges compared with the other animal species.

Increased heart rate was observed in treated camels (Figure 3). Acepromazine produced a similar increase in heart rate in monogastric animals such as cats (0.05 mg/kg) (20) and horses (0.1 mg/kg) (11). Tachycardia and hypotension seem to be conserved features of acepromazine use in ruminant and non-ruminant animals. In cow calves, observed tachycardia and hypotension (19) were possibly due to the α-adrenergic-blocking actions of acepromazine. It also decreases peripheral vascular resistance due to its blockade of the α-adrenergic receptor, leading to peripheral vasodilatation and hypotension, inducing tachycardia (30). In equines, peripheral vasodilatation and hypotension were induced by acepromazine at 0.1 mg/kg, which was reversible by norepinephrine (6).

Previous reports on acepromazine clinical studies in camels did not show a significant change in rectal temperature or respiration rate (15), contrasting with our findings of lowered temperatures and respiratory rates (possibly due to differences in clinical evaluation instruments). The mild hypothermic effect we observed in camels could be attributed to the dilatation of the peripheral blood vessels due to the blockade of α-adrenoceptors. In addition, acepromazine directly depresses the thermoregulatory center in the hypothalamus (3). The experiment was performed at an ambient temperature of 25°C, thereby imposing minimal temperature stress on animals, and avoiding excessive heat stress or cold adaptations. Similar to camels, acepromazine was previously shown to induce a non-significant lower body temperature in cats (1) but a significant decrease in rectal temperature after 1 h in sheep (31). Regarding the respiratory rate, acepromazine induced an initial decrease and then a gradual increase in horses (11) and an overall 36% reduction in pregnant cattle (7).

We observed a significant decrease in hemoglobin, hematocrit, and WBC counts with acepromazine administration. A previous study in camels showed that phenothiazine group drugs caused consistent but statistically insignificant decreases in the hemoglobin concentration and erythrocyte counts 1 h after treatment and induced significant hyperglycemia (15). There were no observed changes in the blood parameters in cow calves (19) and cats (20). Hematocrit, WBC count, RBC count, and hemoglobin % were significantly decreased for 1–8 h after IM injection of horses with 25 mg acepromazine (9). The clinical significance of acepromazine decreasing the hematocrit values seems to be insignificant in healthy animals. However, clinicians should consider the blood parameters of animals receiving acepromazine treatment as weak animals with poor hematological parameters and low hematocrits might be affected by its hematocrit-lowering properties.

Acepromazine induced a significant 55% decrease in hematocrits from 30.00 ± 2.37 to 16.52 ± 11.62 in camels. A previous study in equines revealed that hematocrit was the most sensitive blood parameter after acepromazine application reduced hematocrits by 20% at a dose rate of 0.15 mg/kg (13). In pregnant cattle, an approximately 5% reduction was observed after 45 min (7). There was a similar decrease in PCV in cattle at a dose of 0.05 mg/kg (27). This result might be associated with the sequestration of RBCs into the liver and spleen after acepromazine injection (32). The observed increase in total camel WBCs after acepromazine injection, mainly due to more neutrophils and monocytes, contrasts with reports in other species, in which injection with acepromazine reduced the blood leukocyte count (9). However, the significant decrease in total lymphocyte count and the two T-cell subsets, CD4-positive helper T cells, and WC1-positive gamma delta T cells, indicates a negative effect of acepromazine on camel cellular immunity. These data are also supported by the increased NLR that occurred after injection with acepromazine, as a high NLR has been linked to impaired immune cell function (33, 34). In addition, the decrease in MHC-II expression together with increased abundance of CD163 on blood monocytes after injection with acepromazine suggests the development of an anti-inflammatory phenotype of monocytes (35). However, it is possible that the inhibitory properties of acepromazine on immune cells are a part of its anti-inflammatory action. In this context, acepromazine (0.1 mg/kg) was recommended for inflammatory diseases in equines due to its anti-inflammatory and antioxidant actions (21).

The limitations of this study comprise the use of acepromazine in normal camels, as diseased camels may respond differently. A second issue that requires consideration in future work is that a significant fraction of acepromazine binds with RBCs. Camels have a specific nucleated, oval form of RBC that is different from other animals. It will be important to investigate the contribution of these unique RBCs to the observed aberrant kinetics in camels. Securing camels in sternal recumbency, practiced in this study, to obtain accurate health parameters might impose changes in the measured parameters due to stress on the animals. Furthermore, the influence of acepromazine on immunity is likely to be transient because of the brief duration or single-dose injection. Finally, as there were variable sedation scores with obviously delayed sedation, the extension of clinical exam time, and a dose titration experiment are needed to correlate the dose level of acepromazine with the onset, duration, and progression pattern of sedation in camels.

In conclusion, in evaluating the association between the pharmacokinetic and clinical aspects of acepromazine injection in camels at its recommended clinical dosage of 0.1 mg/kg IV, acepromazine did not produce deep sedation, and the sedation score was in the range of 1–2 for most of the experimental time. The combined clinical and pharmacokinetic parameters indicated the suitability of acepromazine for longer-term sedation, and the sedation profile is suitable for transportation and will minimize animal responses during handling and manipulation without excessive loss of motor functions and coordination.

## Data Availability Statement

The original contributions presented in the study are included in the article/supplementary material, further inquiries can be directed to the corresponding author/s.

## Ethics Statement

The animal study was reviewed and approved by The Ethics Committee of King Faisal University (approval no. KFU-REC/2020-03-02).

## Author Contributions

MK: designed research, performed research, collected data, analyzed data, and wrote the paper. AA: performed research, collected data, and analyzed data. JH: performed research and analyzed the data. WE-D: analyzed data. KV: designed research, performed research, collected data, and analyzed data. All authors revised the paper and approved submission.

## Conflict of Interest

The authors declare that the research was conducted in the absence of any commercial or financial relationships that could be construed as a potential conflict of interest.

## Publisher's Note

All claims expressed in this article are solely those of the authors and do not necessarily represent those of their affiliated organizations, or those of the publisher, the editors and the reviewers. Any product that may be evaluated in this article, or claim that may be made by its manufacturer, is not guaranteed or endorsed by the publisher.
